# Inhibition of carbohydrate hydrolyzing enzymes by a potential probiotic *Levilactobacillus brevis* RAMULAB49 isolated from fermented *Ananas comosus*

**DOI:** 10.3389/fmicb.2023.1190105

**Published:** 2023-06-14

**Authors:** Reshma Mary Martiz, Chandana Kumari V. B., Sujay S. Huligere, Mohd Shahnawaz Khan, Nouf Omar Alafaleq, Saheem Ahmad, Firoz Akhter, Navya Sreepathi, Ashwini P., Ramith Ramu

**Affiliations:** ^1^Department of Biotechnology and Bioinformatics, JSS Academy of Higher Education and Research, Mysuru, Karnataka, India; ^2^Department of Microbiology, JSS Academy of Higher Education and Research, Mysuru, Karnataka, India; ^3^Department of Biochemistry, College of Sciences, King Saud University, Riyadh, Saudi Arabia; ^4^Department of Biosciences, Integral University, Lucknow, India; ^5^Department of Biomedical Engineering, Stony Brook University, Stony Brook, NY, United States

**Keywords:** fermented pineapple, probiotic, α-amylase, α-glucosidase, antidiabetic

## Abstract

The research aimed to explore the potential probiotic characteristics of *Levilactobacillus brevis* RAMULAB49, a strain of lactic acid bacteria (LAB) isolated from fermented pineapple, specifically focusing on its antidiabetic effects. The importance of probiotics in maintaining a balanced gut microbiota and supporting human physiology and metabolism motivated this research. All collected isolates underwent microscopic and biochemical screenings, and those exhibiting Gram-positive characteristics, negative catalase activity, phenol tolerance, gastrointestinal conditions, and adhesion capabilities were selected. Antibiotic susceptibility was assessed, along with safety evaluations encompassing hemolytic and DNase enzyme activity tests. The isolate's antioxidant activity and its ability to inhibit carbohydrate hydrolyzing enzymes were examined. Additionally, organic acid profiling (LC-MS) and *in silico* studies were conducted on the tested extracts. *Levilactobacillus brevis* RAMULAB49 demonstrated desired characteristics such as Gram-positive, negative catalase activity, phenol tolerance, gastrointestinal conditions, hydrophobicity (65.71%), and autoaggregation (77.76%). Coaggregation activity against *Micrococcus luteus, Pseudomonas aeruginosa*, and *Salmonella enterica* serovar Typhimurium was observed. Molecular characterization revealed significant antioxidant activity in *Levilactobacillus brevis* RAMULAB49, with ABTS and DPPH inhibition rates of 74.85% and 60.51%, respectively, at a bacterial cell concentration of 109 CFU/mL. The cell-free supernatant exhibited substantial inhibition of α-amylase (56.19%) and α-glucosidase (55.69%) *in vitro*. *In silico* studies supported these findings, highlighting the inhibitory effects of specific organic acids such as citric acid, hydroxycitric acid, and malic acid, which displayed higher Pa values compared to other compounds. These outcomes underscore the promising antidiabetic potential of *Levilactobacillus brevis* RAMULAB49, isolated from fermented pineapple. Its probiotic properties, including antimicrobial activity, autoaggregation, and gastrointestinal conditions, contribute to its potential therapeutic application. The inhibitory effects on α-amylase and α-glucosidase activities further support its anti-diabetic properties. *In silico* analysis identified specific organic acids that may contribute to the observed antidiabetic effects. *Levilactobacillus brevis* RAMULAB49, as a probiotic isolate derived from fermented pineapple, holds promise as an agent for managing diabetes. Further investigations should focus on evaluating its efficacy and safety *in vivo* to consider its potential therapeutic application in diabetes management.

## Introduction

Diabetes mellitus is a metabolic syndrome characterized by hyperglycemia, and it is caused by a disturbed metabolism because of the ingested food, resulting in a lack of insulin secretion or insulin resistance (Ramu et al., 2014; Sreepathi et al., 2022). Multiple pharmacological approaches have been derived to reduce hyperglycemia with various targets and modes of action, which include inhibition of gluconeogenesis, insulin injection, inhibition of glucose absorption, inhibition of carbohydrate hydrolyzing enzymes, and an increasing number of glucose transporters (Forouhi et al., 2018; Patil et al., 2022a). Among these approaches, inhibition of the carbohydrate hydrolyzing enzymes α-glucosidase and α-amylase has been the most effectively used therapeutic antidiabetic drugs. α-amylase and α-glucosidase are carbohydrate digestive enzymes present in the brush border layer of the small intestine, and both enzymes result in a level-up of glucose in the bloodstream (Etxeberria et al., 2012; Ramu et al., 2016a). The dysregulation between carbohydrate hydrolyzing enzymes and insulin hormone effectively leads to diabetes. During the dysregulation condition, a hyperglycemic state persists for an extended period of time, leading to several associated complications such as angiopathy, neuropathy, nephropathy, and retinopathy (Telagari and Hullatti, 2015). Although therapeutic drugs are effective in the management of postprandial hyperglycemia, their consumption for a long term is often associated with multiple adverse effects. These side effects have further driven the researchers to seek an effective therapy with fewer adverse effects (Chaudhury et al., 2017; Ramu and Patil, 2021).

Consumption of food that replaces medication promotes health status and reduces the risk of the disease. A previous study proposed a new approach to nutrition science to treat the condition and addressed in parallel the increasing life expectancy, promoting an improved quality of life (Patil et al., 2021a). In this context, functional foods such as probiotics have received attention in healthcare systems. Functional foods are those that act as traditional nutrients and are accompanied by other beneficial effects such as preventing nutrition-related diseases (Nguyen et al., 2019; Ramu et al., 2022).

For the treatment of hyperglycemia, probiotics are an alternative reliable approach used to inhibit carbohydrate hydrolyzing enzymes and monitor gut health (Ayyash et al., 2018). As microorganisms colonizing the gut (called gut microbiota) play a vital role in physiology and metabolism, they are expected to play a pivotal role in rendering health-promoting effects (Gérard and Vidal, 2019). It is a well-known fact that patients with diabetes mellitus have altered gut microbiota; thus, inhibiting carbohydrate hydrolyzing enzymes and monitoring gut health probiotics could be an effective remedy that would produce minimal side effects. Probiotics are also economical in comparison with any other orally consuming synthetic hypoglycaemic drug (Li et al., 2020). Probiotics have effective antihyperglycemic components; nevertheless, for historical and technological reasons, most of the available probiotics are based on dairy products, which may cause some inconvenience to the consumer who are lactose intolerant. Fermented vegetables and fruits were evaluated for their probiotic potential more recently by Nguyen et al. (2019).

*Ananas comosus* (pineapple) is the third most important fruit of the tropical and sub-tropical regions and belongs to the *Bromeliaceae* family. It is a fruit rich in carbohydrates, proteins, vitamins (C, K, A, and B6), riboflavin, thiamine, pantothenic acid, choline, betaine, phytosterols, minerals (calcium, iron, magnesium, phosphorous, potassium, sodium, zinc, copper, and manganese), and phenolic compounds (gallic acid, chlorogenic acid, and ferulic acid; Brat et al., 2004; Mhatre et al., 2009; Nguyen et al., 2019). Pineapple was also found to possess antioxidant, anticarcinogenic, and antimutagenic properties along with a protective role against cataracts and cardiovascular diseases. According to Nguyen et al. (2019), the fermentation of pineapple juice with *Lactobacillus* and *Bifidobacterium* strains of probiotic bacteria demonstrated their ability to survive in a highly acidic environment (Nguyen et al., 2019). Because all of the beneficiary nutrients of pineapple are highly supported by its pleasing taste, the fruit can be considered a unique food matrix to carry probiotics to all age groups with a convincing taste (Perricone et al., 2015). With this background, the objective of the present study was framed to isolate potential lactic acid bacterial strains from fermented pineapple with the high potentiality to inhibit carbohydrate hydrolyzing enzymes.

## Materials and methods

### Materials

All the required chemicals for the present study were purchased from HiMedia Laboratories (Mumbai, India). The pathogens that were used in the study for antibacterial tolerance and coaggregation assay were purchased from Microbial Type Culture Collection and Gene Bank (MTCC), Chandigarh, India.

### Sample preparation and isolation

The fresh medium-ripened pineapple (2 kg) belonging to the “Queen” variety was purchased from a local marketplace during the growing season (June 2022, Mysuru, Karnataka). To maintain a sterile environment, the fruit was washed with lukewarm water. After removing the peel, the fruit was cut into small pieces and rewashed with lukewarm salt water. The fruit pieces (800 g) were added to a glass jar with 3% table salt and left to ferment in two batches for 6 days. To obtain the LAB strains, 1 g of pooled sample was serially diluted and plated on MRS agar on the last day of fermentation. The obtained single distinctive colonies were further inoculated into MRS broth, followed by replating for purification. Colonies were selected for further studies based on Gram staining and catalase test, and glycerol stock was prepared from the same colonies possessing the baseline criteria (Kumari et al., 2022a,b).

### Physiological properties and biochemical characterization of LAB strains

The isolate was further characterized based on the physiological properties to withstand different temperatures (4, 15, 37, and 45°C), pH (2, 4, 6, and 7.4 in 37°C for 24 h), and NaCl concentrations (2, 4, 6, and 10% in 37°C for 24 h) as per Bergey's manual. The ability of the isolate to ferment different monosaccharides and disaccharides was assessed as per the protocol described by Divyashree et al. (2021). Furthermore, biochemical tests such as Voges–Proskauer, gelatin liquefaction, starch hydrolysis, indole, and methyl red tests were performed as mentioned by Huligere et al. (2022).

### Molecular identification

The LAB isolate was subjected to DNA extraction and evaluated through molecular techniques using 16S rRNA gene sequencing with 27F [5′AGA GTTTGATCCTGGCTCAG3′] and 1492R [5′GGTTACCT TGTTACGACTT3′] primers. After sequencing, the sequence was deposited in the GenBank sequence database. The obtained sequence was assessed through the homology search using BLAST. Furthermore, the MEGA X software (version 10.2.4, CA, USA) was used to build a phylogenetic tree with 1,000 bootstraps (Tamura and Nei, 1993).

### Probiotic properties of an LAB strain

#### Phenol tolerance assay

The resistance of the isolate against phenol was determined as per the protocol described by Jena et al. (2013) with some modifications. In brief, the overnight culture of LAB isolate was inoculated into MRS broth with 0.1 and 0.4% phenol, separately, and incubated (0, 4, and 24 h; 37°C). After the incubation period, the viable cell count was calculated by plating the above suspension on an MRS agar plate.

#### Acid and bile salt tolerance

The overnight LAB culture was inoculated into MRS broth (pH 2) with 0.3 and 1% oxgall [HiMedia Laboratories (Mumbai, India)], separately, and incubated for 4 h according to the methodology described in the study by Kumari et al. (2022a,b) with slight modifications. The above suspensions were plated at 0, 2, and 4 h of inoculation to observe the survivability of LAB isolates against acid bile conditions at varying time intervals. The plates were incubated for 24 h at 37°C to understand the survivability of the cells.

#### Cell surface hydrophobicity of LAB isolate

The protocol by Guan and Liu (2020) with slight modifications was used to know the cell surface hydrophobicity of the isolate with polar xylene solvent, which indirectly determines the ability of the isolate to adhere to intestinal cells. The assay was performed in duplicates and repeated thrice, and surface hydrophobicity was computed using the equation below:

#### Autoaggregation and coaggregation ability of the LAB strain

The autoaggregation and coaggregation assay was conducted as previously described by Li et al. (2020) with minor modifications. In brief, for autoaggregation, the overnight culture was harvested by centrifuging at 500 × *g* for 10 min. The obtained pellets were washed thrice with sterile PBS and resuspended in the same PBS to obtain a cell count of 10^8^ CFU/mL. The suspension was incubated at 37°C and at varying time intervals (2, 4, 6, 10, and 24 h). A measure of 100 μL of the topmost layer of the suspension was transferred to a 96-well plate, and the absorbance was read at 600 nm. The rate of autoaggregation was determined by the equation below:


$$\begin{array}{l} \text{Autoaggregation}\left(\%\right)=\frac{{\text{A}}^{\text{z}}-{\text{A}}^{\text{t}}}{{\text{A}}^{\text{z}}}\times \text{1}00 \end{array}$$

A^z^ = absorbance at time zero.

A^t^ = absorbance at time (2, 4, 6, 10, and 24 h).

Coaggregation was performed using LAB pellets suspended in PBS as mentioned in the autoaggregation procedure and mixed with 1 mL of different bacterial suspension (*Pseudomonas aeruginosa* MTCC 424, *Salmonella enterica* serovar Typhimurium MTCC 98, *Bacillus subtilis* MTCC 10403, *Micrococcus luteus* MTCC 1809, and *Escherichia coli* MTCC 4430) and incubated for 120 min at 37°C. After incubation, the absorbance of all the suspensions was read at 600 nm. The below equation was used to calculate the percentage of coaggregation:


$$\frac{[({\text{A}}^{\text{LAB}}{\text{+A}}^{\text{pathogen}})-{\text{A}}^{\text{mixture}}\times \text{100}}{\left[{\text{A}}^{\text{LAB}}{\text{+A}}^{\text{pathogen}}\right]}$$

A^LAB^ + A^pathogen^ = absorbance of LAB and pathogen suspension mixture at 0 h.

A^mixture^ = absorbance LAB and pathogen suspension mixture at 2 h.

#### Survivability at simulated gastric and intestinal fluids

The ability of the LAB isolates to tolerate gastric and intestinal juices was assessed as per the protocol described by Reale et al. (2015) with slight modifications. To provide simulated gastric and simulated intestinal juice conditions, pepsin (3,000 mg/L of PBS, pH 3; 1:3,000 AU/mg, Sisco Research Laboratories Pvt. Ltd., Mumbai, India) and trypsin (1,000 mg/L of PBS, pH 8; 2,000 U/g, Sisco Research Laboratories Pvt. Ltd., India) were dissolved. The isolates were then added to the simulated juices in an *in vitro* environment under gastrointestinal conditions (10^8^ CFU/mL). The equation below was used to calculate the survival rate:


$$\begin{array}{l} \text{Survival rate }(\%)\;=\frac{\left({\text{Log CFU N}}_{\text{a}}\right)}{\left({\text{Log CFU N}}_{\text{b}}\right)}\times \text{1}00 \end{array}$$

N_a_ = number of viable cells after treatment.

N_b_ = number of viable cells before treatment.

#### Antibacterial activity

The antagonistic activity of the isolate was determined by the disk diffusion method as described by Mezaini et al. (2009) with modifications against 10 foodborne pathogens (*Escherichia coli* MTCC 443*, Bacillus subtilis* MTCC 10403*, Micrococcus luteus* MTCC 1809, *Pseudomonas aeruginosa* MTCC 424, *Salmonella enterica* serovar Typhimurium MTCC 98, *Bacillus cereus* MTCC 1272, *Staphylococcus aureus* MTCC 1144, *Klebsiella pneumonia* MTCC 10309, *Pseudomonas fluorescens* MTCC 667, and *Klebsiella aerogenes* MTCC 2822). The results are predicted as good, moderate, weak, and null inhibition based on the size of the clear inhibitory zone observed around the disk.

#### Antibiotic susceptibility test

The antibiotic sensitivity of the LAB isolate was determined by disk diffusion method against 10 antibiotics gentamicin (10 μg), chloramphenicol (30 μg), clindamycin (2 μg), ampicillin (10 μg), kanamycin (30 μg), tetracycline (30 μg), vancomycin (30 μg), erythromycin (15 μg), streptomycin (100 μg), rifampicin (5 μg), methicillin (5 μg), azithromycin (15 μg), and cefixime (5 μg). The diameter of the antibiotic zone was measured using the Clinical and Laboratory Standards Institute (CLSI 2018) scale (Chang, 2018), and the results are predicted as sensitive and resistant (Hana et al., 2015).

#### Adhesion assay

Adhesion assay of the isolate was performed with chicken crop epithelial cells and buccal epithelial cells. Through crystal violet staining and microscopic observation, the adherent ability of the isolate with chicken crop epithelial cells under *in vitro* conditions was assessed as per the protocol described by Somashekaraiah et al. (2019). In brief, about 100 μL (10^8^ CFU/mL) of LAB isolate was mixed with 400 μL of chicken crop cell and incubated for 30 m at 37°C. After incubation and centrifugation (500 × *g*, 5 min), the pellets were washed with PBS twice to remove non-adherent cells. Furthermore, the pellet was resuspended in PBS (100 μL) and stained for microscopic observation. The bacterial adhesion is examined and scored positive if a minimum of 10 LAB cells have adhered with one chicken crop epithelial cell.

The ability of the isolate to adhere to the buccal epithelial cells was studied as per the protocol described by Kumari et al. (2022a,b). In brief, the buccal epithelial cells were collected from a volunteer after rinsing the mouth with saline to avoid microbiota. The collected cells were washed with saline and centrifuged (500 × *g*, 5 min), and the pellets were washed with PBS and resuspended in saline. About 400 μL of buccal epithelial cells were mixed with 100 μL (10^8^ CFU/mL) of LAB isolate and incubated for 30 min. After crystal violet staining, through microscopic observation, the adhesion of the isolate with the buccal epithelial cell was determined.

#### Hemolytic and DNase activity of the LAB isolate

The two safety assessment assays, namely, hemolytic and DNase activity, were performed as per the protocol described by Li et al. (2020) and Somashekaraiah et al. (2021), respectively. In brief, the hemolytic activity of the isolate was determined by streaking LAB isolate on a blood agar medium containing sheep blood (5% w/v) and incubated at 37°C for 24 h. After incubation, the plates were examined for α-hemolysis, β-hemolysis, and γ-hemolysis (no zone formation around colonies) activities.

The ability of the LAB isolates to produce DNase enzyme was determined by streaking LAB isolate on the DNase agar medium and incubating it at 37°C for 24 h. The formation of the pink zone around the colonies indicates that the isolate is positive for the DNase enzyme.

#### Antioxidant activity

The scavenging effect of the LAB isolates on ABTS and DPPH was performed as per the protocol described by Huang et al. (2019) and Li et al. (2012) with slight modifications. The below-mentioned equation was used to compute the scavenging activity of the isolate:


$$\begin{array}{l} \text{ABTS scavenging activity }(\%)\;=\frac{{\text{A}}_{\text{c}}-{\text{A}}_{\text{s}}\pm {\text{A}}_{\text{m}}}{{\text{A}}_{\text{c}}}\times \text{1}00\\ \text{ DPPH scavenging activity }(\%)\;=\frac{{\text{A}}_{\text{c}}-{\text{A}}_{\text{s}}}{{\text{A}}_{\text{c}}}\times \text{1}00 \end{array}$$

A_c_ = absorbance of control.

A_s_ = absorbance of the sample.

A_m_ = absorbance of the mixture (sample with control).

#### α-glucosidase and α-amylase inhibition

The ability of the isolate to inhibit α-glucosidase and α-amylase was determined as per the protocol described by Maradesha et al. (2022a) and Banu et al. (2023) with slight modifications. In this assay, the inhibitory activity of intact cells (I), cell-free extract (E), and cell-free supernatant (S) was prepared as per the description by Kumari et al. (2022a,b). Test samples I, E, and S were combined with 50 mM of potassium phosphate buffer (pH 6.8, 700 μL) and then they were left for 10 min. It was then pre-incubated at 37°C for 15 min with the enzyme α-glucosidase (100 μL, 0.25 U/mL). Then, 100 μL of 5 mM p-nitrophenol-D-glucopyranoside (pNPG) substrate was added. The reaction was then stopped with 1,000 μL of 0.1 M Na_2_CO_3_ after 30 min of incubation at 37°C. Using a microplate reader, the 4-nitrophenol absorption was measured at 405 nm (Multiskan FC Microplate Photometer, Thermo Fisher Scientific, France), and the inhibition of the α-glucosidase activity of LAB strain was calculated as below:


$$\begin{array}{l} \text{Inhibition of α}-\text{glucosidase }(\%)=(1-{\text{S}}^{\text{A}}/{\text{C}}^{\text{A}})\times 100 \end{array}$$

where, S^A^ = absorbance of the reactants with the sample and C^A^ = absorbance of the reactants without the sample.

The amylase inhibitory assay utilized porcine pancreatic amylase. In a brief, 500 mL of I, E, and S were pre-incubated for 10 min at 25°C with 500 mL of 0.1 M PBS (pH 7.4) containing α-amylase enzyme (0.5 mg/mL). Additionally, 500 mL of a 1% starch solution in 0.1 M PBS were added to each tube (pH 7.4). The reaction solutions were then stopped with 1.0 mL of the 3,5-dinitro salicylic acid reagent after being incubated for 10 min at 25°C. After 5 min in a boiling water bath, the test tubes were cooled to room temperature, diluted with 10 mL of distilled water, and the absorbance was measured at 540 nm. The percentage of inhibition exerted by the bacterial strain on α-amylase activity was obtained as defined for α-glucosidase.

#### Extraction of organic acid

The inhibitory activity of cell-free supernatant of LAB against carbohydrate hydrolyzing enzymes was further analyzed by extracting organic acids. The sample preparation for extraction was performed as per Lee et al. (2012) with slight modifications. In brief, 5 mL of MRS broth in screw cap test tubes was inoculated with 0.5 mL of previously activated LAB isolate and incubate at 37°C for 5 days in a shaking incubator (150 rpm). After incubation, the poured samples were centrifuged at 5,590 × *g* for 20 min at 4°C. The obtained sample was then used for LC-MS analysis to extract organic acids (Ramu et al., 2015; Pushpa et al., 2022).

### *In silico* studies

#### Pass pharmacological analysis

The pharmacological activity of the analyzed secondary metabolites was evaluated by PASS (online server). The server evaluated the potentialities of the input sample and specifies its pharmacological impacts (Patil et al., 2022b). The whole collected data were computed and classified as probable active and inactive and denoted as Pa and Pi, respectively. The compound with the Pa value greater than Pi was considered probable active with a particular pharmacological activity (Patil et al., 2022c).

#### Molecular docking simulation

For molecular docking simulation, the protein preparation (α-glucosidase and α-amylase) was performed according to the protocol described by Maradesha et al. (2022b). Furthermore, the binding site prediction for both the target proteins was performed based on the literature survey. The grid box size of both α-glucosidase and α-amylase targets was 40 Å × 40 Å × 40 Å positioned at the coordinates x = −17.489 Å, y = −8.621 Å, z = −19.658 Å and x = 103.469 Å, y = 37.176 Å, z = 19.607 Å, respectively (Martiz et al., 2022a). Concurrently, the ligand structures of lactic acid, pyruvic acid, malonic acid, maleic acid, fumaric acid, succinic acid, malic acid, tartaric acid, shikimic acid, citric acid, and hydroxycitric acid was optimized into 3D models using ACD ChemSketch (Patil et al., 2021b). Currently, the prepared ligand and protein were docked using AutoDock Vina 1.1.2 with acarbose as the positive control (Gurupadaswamy et al., 2022; Jyothi et al., 2022).

#### Molecular dynamics (MD) simulations

After the docking study, the compound with the best binding affinity, hydrogen bond, and the number of interactions was screened and considered the lead compound. An MD simulation was performed for this compound to understand the binding stability and conformational changes that take place during the complex formation (Patil et al., 2022d,e). The simulation was performed for a 100 ns timescale using the biomolecular software package GROMACS-2018.1. The pdb2gmx program protein was assigned with CHARMM36 force field to obtain protein topology, and the ligand topology was obtained by the SwissParam server (Kumar et al., 2021). To maintain neutrality and salt concentration (0.15 M) of the entire system, the counter Na+ and Cl- ions were supplied. Furthermore, energy minimization was performed using the steepest descent method of 50,000 steps, and the system was equilibrated in two phases, NVT and NPT ensemble (1,000 ps each), with a 310 K temperature and 1 bar pressure (Kumar et al., 2022). After MD simulation, the trajectories were analyzed, and a graph was generated for the RMSD, RMSF, Rg, SASA, and hydrogen bond using xmgrace software (Maradesha et al., 2023).

#### Binding free energy calculations

Using the g_mmpbsa program, which is a GROMACS plugin, the binding free energy of the complex was estimated by employing the Molecular Mechanics Poisson–Boltzmann Surface Area (MM-PBSA) method. MM-PBSA was quantitatively evaluated in accordance with the research done by Martiz et al. (2022b). The binding free energy is calculated using three components: molecular mechanical energy, polar and apolar solvation energies, and molecular mechanical energy. The calculation was performed for the last 50 ns frames, which were extracted from MD trajectory data (Martiz et al., 2022c).

### Statistical analysis

All the experiments were carried out in triplicates, and the standard deviation is displayed in error bars on the graph. Data were examined using ANOVA, and differences were considered substantial at a *p*-value of ≤ 0.05 (Ramu et al., 2014, 2016b).

## Results

Initially, several colonies from the fermented pineapple sample were isolated and selected six colonies that showed distinct morphological characteristics. Among the colonies, the isolate RAMULAB49 was chosen because it was a rod-shaped, Gram-positive, catalase-negative bacterium with optimal growth at 37°C and demonstrated tolerance for pH values ranging from 2 to 6, with 7.4 being considered the ideal pH. Additionally, the isolate demonstrated tolerance for NaCl concentrations as high as 4%. The biochemical characteristics of the isolate revealed its heterofermentative nature, and its fermentation of carbohydrates did not produce any gas (Table 1).

**Table 1:** Preliminary tests, phenotypic characterization, biochemical properties, and fermentation ability of the LAB strain isolated from fermented pineapple.

	**Test**	Isolate^*^

		RAMULAB49
Preliminary tests	Gram staining	Positive
	Catalase	Negative
	Morphology	Rod
Temperature tolerance (°C)	4	-
	15	-
	37	+
	45	-
pH	2	+
	4	+
	6	+
	7.4	+
NaCl tolerance (%)	2	+
	4	+
	6	-
	10	-
Biochemical tests	Citrate utilization test	-
	Gelatin liquefaction test	-
	Voges-Proskauer test	-
	Starch hydrolysis test	-
	Indole test	-
	Methyl red test	+
Carbohydrate fermentation	Lactose	+
	Maltose	+
	Sucrose	+
	Lactose	+
	Arabinose	+
	Fructose	+
	Sorbitol	+
	Mannitol	+
	Galactose	+
	Xylose	-
	Glucose	+

^*^”+” indicates presence and “-“ indicates absence.

### Molecular character and phylogenetic analysis

**Figure 1:**
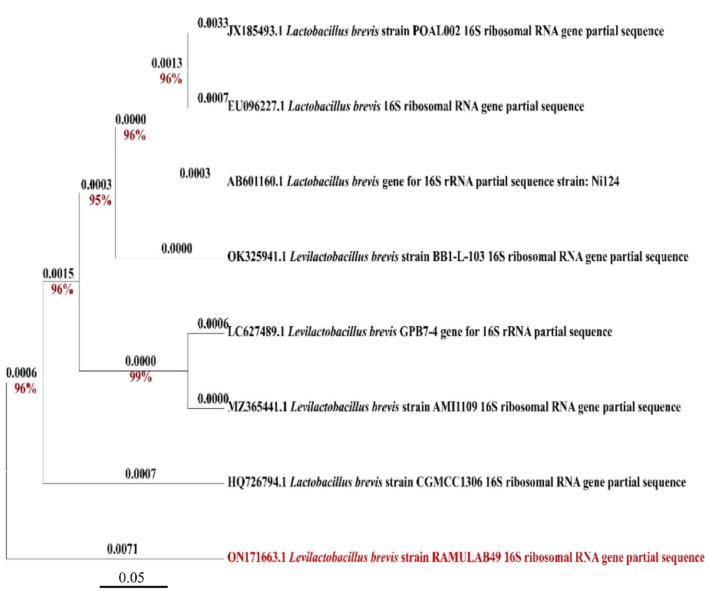
Phylogenetic tree of RAMULAB49 (*Levilactobacillus brevis*) isolated from fermented pineapple based on the maximum likelihood bootstrap analysis of 16S rDNA.

With a similarity of 98.83%, *Levilactobacillus brevis* was determined to be the LAB isolate obtained in this study and termed RAMULAB49 based on 16S rRNA sequencing and evolutionary investigations by MEGA X (Version 10.2.4, CA, USA). MEGA X was used to construct a phylogenetic tree using other additional reference strains from different sources, which produced findings that were equivalent and indicated that the additional strains are members of the same *Lactobacillaceae* family. The GenBank accession number for the partial sequence was ON171663, which was obtained after submission to NCBI in Figure 1.

### Probiotic properties of LAB isolate

#### Phenol tolerance

The graph indicates that the isolate exhibited phenol tolerance. The viable count of the isolate, assessed at a higher phenol concentration of 0.4% during the last hour of incubation, was determined to be 7.42 log CFU/mL. Distinct variations in growth rate were observed at 0.4% phenol concentrations but different incubation intervals (0, 4, and 24 h; Table 2).

**Table 2:** Viable count of the RAMULAB49 under phenolic conditions (0.1 and 0.4%).

	**Phenol tolerance (Log CFU/mL)** ^*^	
	**0.1%**	**0.4%**
0	8.6624 ±0.1^c^	7.8006 ± 0.2^c^
4	8.5612 ± 0.5^b^	7.4532 ± 0.4^b^
24	8.4635 ± 0.2^a^	7.4206 ± 0.1^a^

^*^Data are expressed in mean ± SD with significantly different P-values (P ≤ 0.05), which are represented with superscripts (a–c) and separated by the Duncan-multiple range test.

#### Acid bile salt tolerance

**Figure 2:**
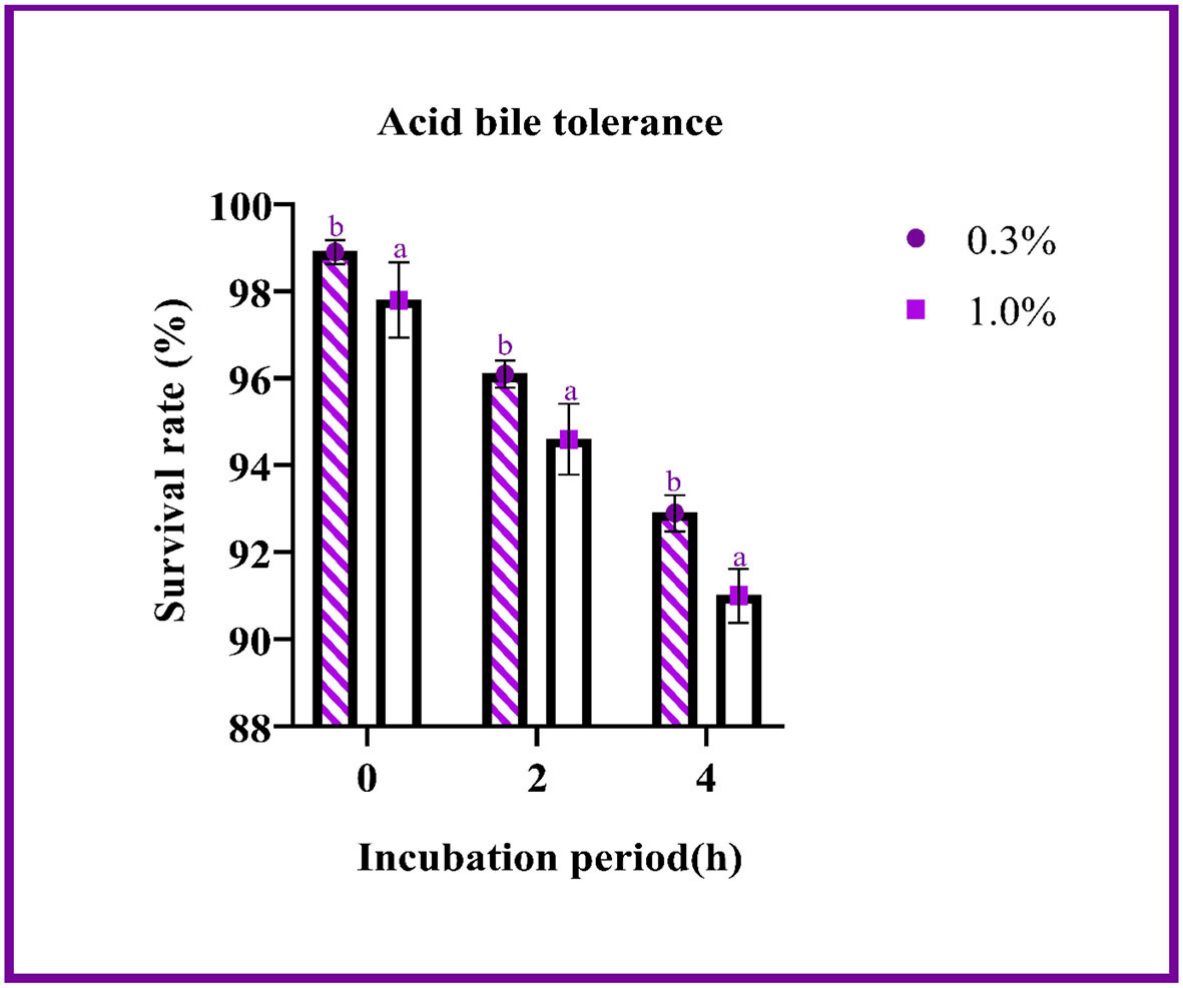
The survival rate of the LAB isolates under acid bile conditions (0.3 and 1.0%) is expressed as mean ± SD with significantly different *P*-values (*P* ≤ 0.05), which is represented with a and b superscripts, and separated by the Duncan-multiple range test.

The ability of the LAB isolates to withstand acid bile conditions was determined with this assay. Figure 2 represents the survival rate of the isolate at pH 2 with 0.3 and 1% oxgall concentration. The difference in survival between the various concentrations was found to be 1% at 4 h, and the survival rate was determined to be above 90%. The probability of survival did not change significantly with changes in oxgall concentration.

#### Cell surface hydrophobicity

The hydrophobic elements of the outer membrane are present in the LABs and serve as the foundation for the cell surface's hydrophobicity. For LAB, hydrophobicity was used to assess isolate colonization and cell adhesion to epithelial cells. The strain RAMULAB49 hydrophobicity to xylene (a non-polar solvent) was discovered to be 65.71 ± 0.22%. Higher hydrophobicity in a strain promotes greater colonization of the intestinal lining.

#### Autoaggregation and coaggregation

An exponential rate of autoaggregation was observed with a progression in the incubation time. At the end of the incubation period, an autoaggregation of 77.76 ± 3.09% was observed. The isolate expressed the highest percentage of coaggregation with *M. luteus* (21.98 ± 0.18%) and the lowest percentage of coaggregation with *Salmonella enterica* serovar Typhimurium (15.72 ± 0.11%). For bacterial colonization and defenses, the probiotics' capacity to autoaggregate and coaggregate is essential (Table 3).

**Table 3:** Autoaggregation and coaggregation abilities of the LAB strain isolated from fermented pineapple.

**Autoaggregation**		**Coaggregation**	
**Incubation time (h)**	**Autoaggregation (%)** ^*^	**Pathogens**	**Coaggregation (%)** ^*^
2	29.87 ± 0.71^a^	*Escherichia coli*	19.14 ± 0.17^d^
4	39.09 ± 0.52^b^	*Micrococcus luteus*	21.98 ± 0.19^e^
6	50.66 ± 0.77^c^	*Bacillus subtilis*	17.32 ± 0.15^c^
10	69.36 ± 0.46^d^	*Salmonella enterica* serovar Typhimurium	15.72 ± 0.11^a^
24	77.76 ± 0.91^e^	*Pseudomonas aeruginosa*	16.06 ± 0.19^b^

^*^The values are shown as mean ± SD. According to Duncan's multiple range test, means in the same column denoted by various letters (a–e) are substantially different (P ≤ 0.05).

#### Survivability at simulated gastric and intestinal fluids

**Figure 3:**
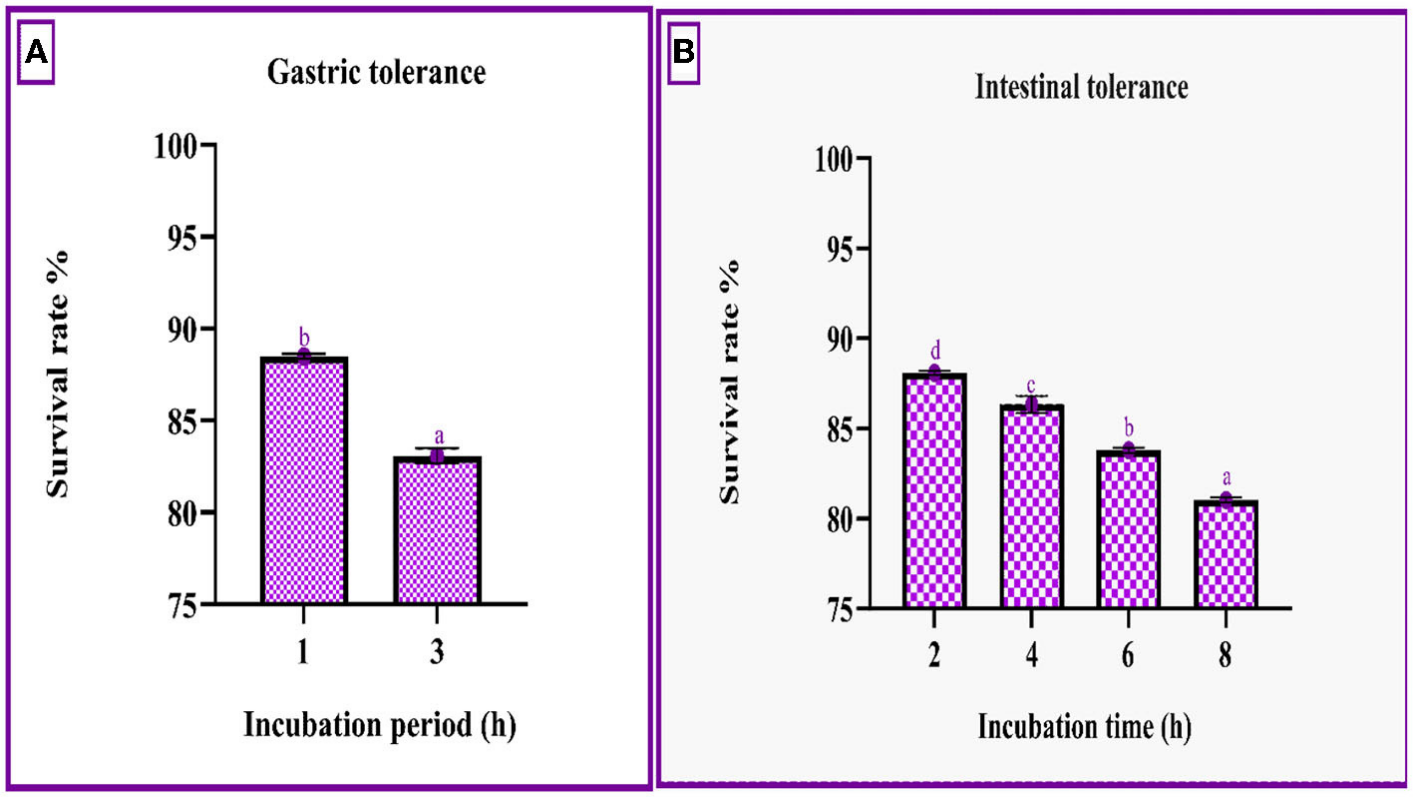
Gastric **(A)** and intestinal juice **(B)** survivability of RAMULAB49 strains after incubation for various time intervals at 37°C. The values are expressed as mean ± SD with significantly different *P*-values (*P* ≤ 0.05), which are represented with superscripts and separated by the Duncan-multiple range test (a–d).

Figure 3A demonstrates that the strain had a survival rate of more than 80% at various incubation intervals of simulated gastrointestinal conditions. The gastric survival was determined to be 88.49 ± 0.12% during the initial incubation period and decreased by 5% after 3 h of incubation. The strain's intestinal survivability decreased with time (~2%) as the incubation period prolonged, as shown in Figures 3A, B.

#### Antibacterial activity

The antibacterial activity of the isolate RAMULAB49 from fermented pineapple was tested against 10 food-borne pathogens, revealing varying degrees of inhibition in the resulting zone. The inhibition zone varied between 5 and 18 mm (Table 4).

**Table 4:** Antibacterial activity of the LAB strain isolated from fermented pineapple.

**Pathogens**	Inhibition activity^*^
*M. luteus*	+++
*P. aeruginosa*	+++
*Salmonella enterica* serovar Typhimurium	+++
*Bacillus subtilis*	++
*Escherichia coli*	++
*Staphylococcus aureus*	++
*Bacillus cereus*	++
*Klebsiella pneumonia*	-
*Klebsiella aerogenes*	++
*Pseudomonas fluorescens*	+

^*^Symbols represent the type of inhibition: (+++) good inhibition 15–18 mm, (++) moderate inhibition 9–15 mm, (+) weak inhibition 3–9 mm, and (-) null inhibition.

#### Antibiotic susceptibility test

To determine the antibiotic sensitivity, the isolate was screened against 10 different antibiotics, and the results were compared with a standard reference chart. The assessment of antibiotic sensitivity of the LAB isolates can be considered one of the basic criteria to be qualified as a potent probiotic agent. In this study, the LAB strain was found to be sensitive to chloramphenicol, clindamycin, streptomycin, cefixime, gentamicin, ampicillin, tetracycline, erythromycin, and azithromycin and resistant to kanamycin, vancomycin, methicillin, and rifampicin (Table 5).

**Table 5:** Antibiotic susceptibility of RAMULAB49 against 10 antibiotics.

**Antibiotic**	Susceptibility^*^
Chloramphenicol	S
Clindamycin	S
Kanamycin	R
Vancomycin	R
Streptomycin	S
Methicillin	R
Cefixime	S
Gentamicin	S
Ampicillin	S
Tetracycline	S
Erythromycin	S
Rifampicin	R
Azithromycin	S

^*^The letter (S) represents sensitivity and (R) represents resistance.

#### Adhesion assay

One of the most important criteria for choosing a probiotic is the adhesion of bacteria to mucosal surfaces and epithelial cells. Pathogen adherence can be prevented by probiotic strains by competing for the binding sites on the host cell. It was discovered that 20–45 bacterial cells could adhere to chicken crop epithelial cells. The isolate also demonstrated adhesion with buccal epithelial cells, with an average of 32–57 bacterial cells adhering to each epithelial cell.

#### Hemolytic and DNase activity

Hemolytic and DNase activities are the two tests that can be used to predict if a LAB isolate will be harmful when administered as a probiotic. After 24 h of incubation, as no zone formation was observed around the bacterial colonies, the LAB isolate is considered safe and categorized as γ-hemolysis. A DNase activity assay also expressed no zone, which means that the LAB strain used in the present study is negative for DNase enzyme activity, non-pathogenic by nature, and safe as a probiotic.

#### Antioxidant activity

**Figure 4:**
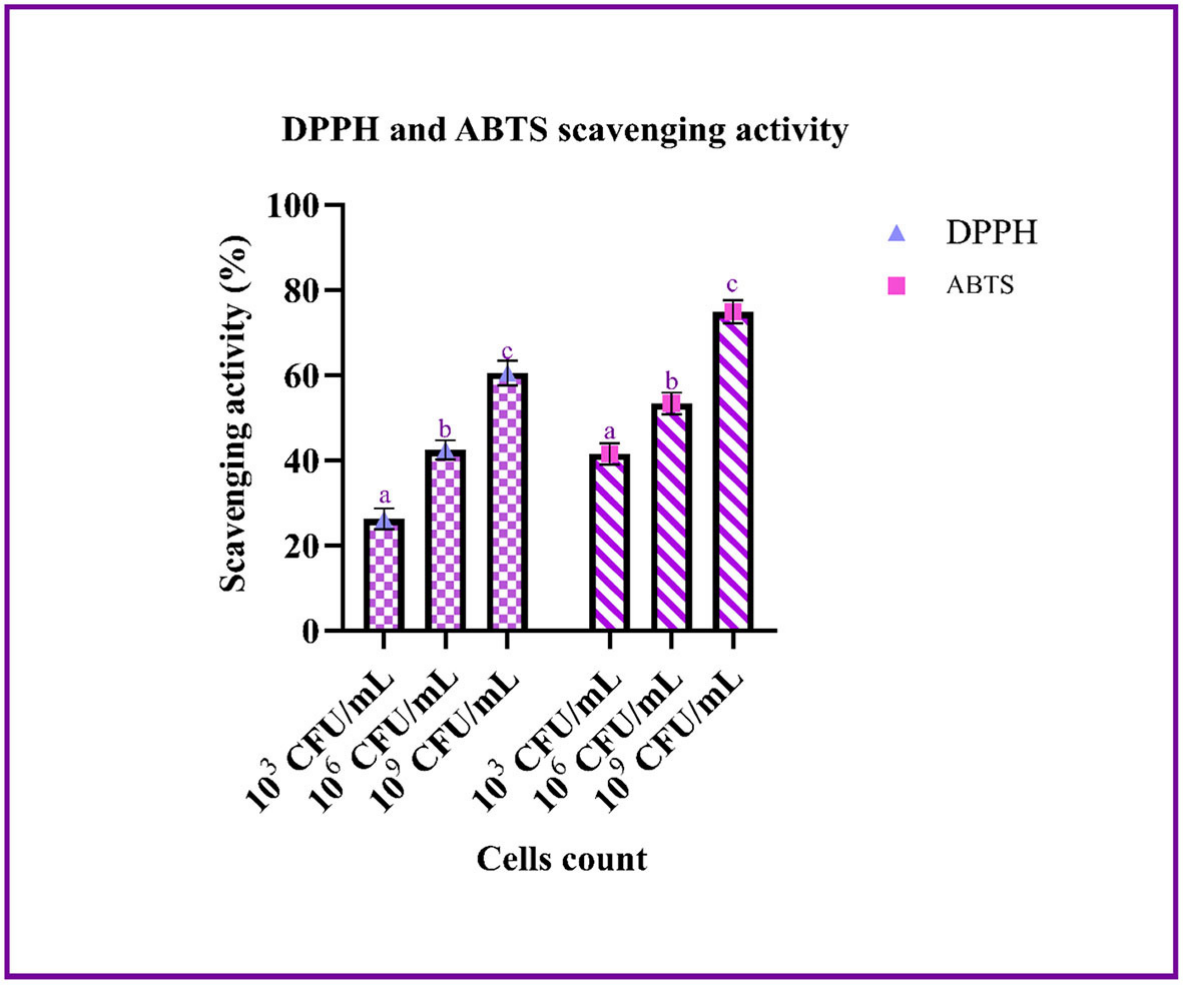
The antioxidant ability of the LAB isolates (10^3^, 10^6^, and 10^9^ CFU/mL) against ABTS and DPPH is expressed in mean ± SD with significantly different *P*-values (*P* ≤ 0.05), which is represented with a, b, and c superscripts and separated by the Duncan-multiple range test.

The scavenging effect of the LAB strain on ABTS and DPPH was performed using a different count of cells. From the graph, it can be inferred that the scavenging activity increases with an increase in cell count. The highest scavenging activity of ABTS and DPPH was found to be 74.85 ± 0.75 and 60.51 ± 0.41%, respectively, with 10^9^ CFU/mL of bacterial cells (Figure 4).

#### α-glucosidase and α-amylase inhibition

**Figure 5:**
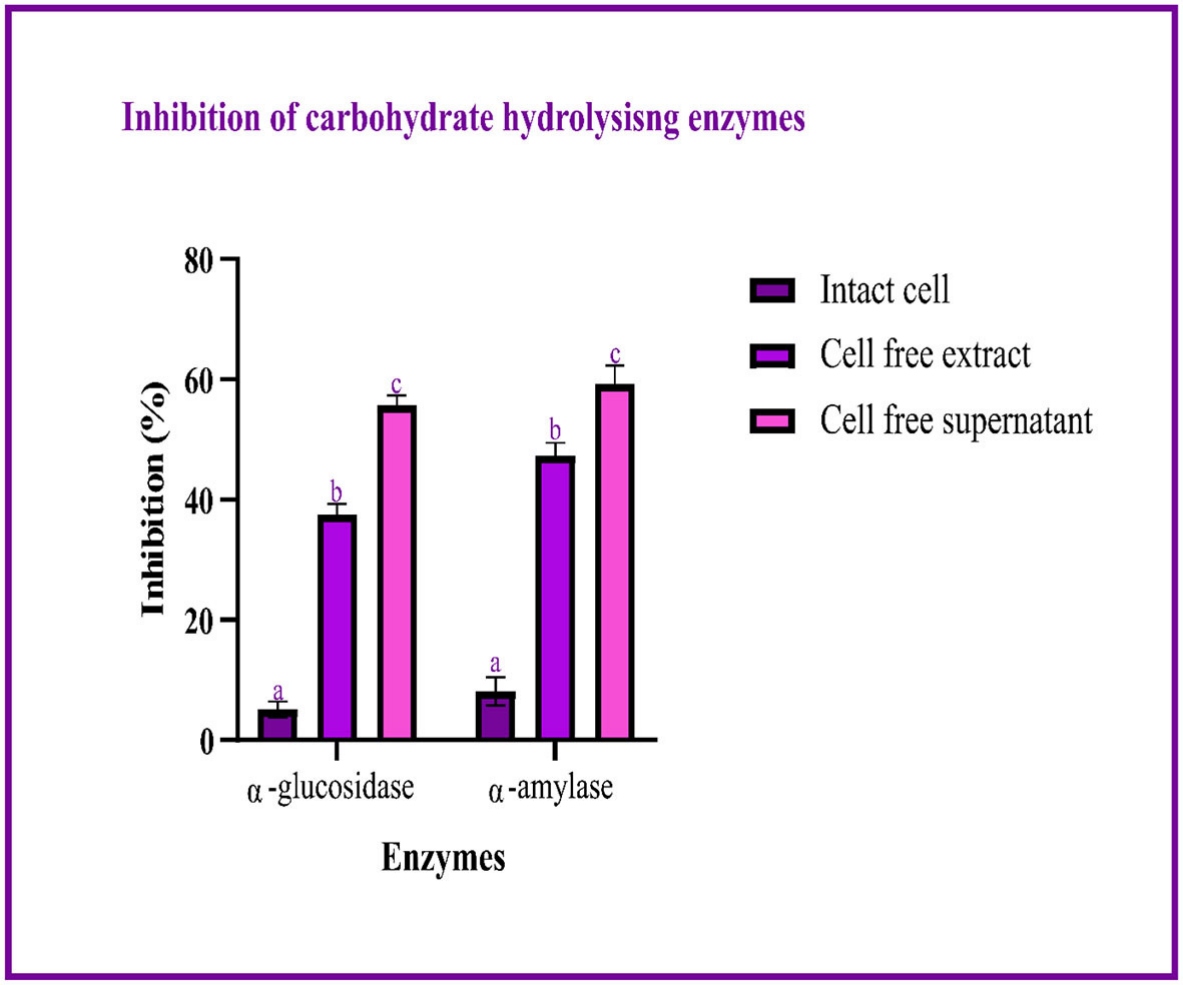
Inhibitory activity of LAB isolates (I, E, and S) against carbohydrate hydrolyzing enzymes is expressed in mean ± SD with significantly different *P*-values (*P* ≤ 0.05), which are represented with a, b, and c superscripts and separated by the Duncan-multiple range test.

The study used intact cells, a cell-free extract, and the cell-free supernatant to measure the inhibitory activity against α-glucosidase and α-amylase. The highest percentage of inhibition is expressed by the cell-free supernatant. The inhibition of α-glucosidase and α-amylase ranged between 5.08–55.69 and 8.1–56.19%, respectively, with intact cells, the cell-free extract, and the cell-free supernatant, as shown in Figure 5.

#### Organic acids profile

The major function of any probiotic strain is to metabolize the sugar present in its media to produce lactic acid and secondary metabolites such as organic acids. The organic acids listed in Table 6 were observed by LC-MS analysis. In this experiment, the cell-free supernatant of RAMULAB49 strains showed higher inhibition against carbohydrate hydrolyzing enzymes (α-glucosidase and α-amylase). Thus, the cell-free extract was subjected to the organic acid profile, which showed that succinic acid is present in large amounts in comparison to the other organic acids listed in Table 6.

**Table 6:** Organic acids' profile obtained by the LC-MS analysis.

**Organic acids**	**RAMULAB49 (mg/mL)**
Lactic acid	1.36453
Pyruvic acid	1.38166
Malonic acid	0.04465
Maleic acid	0.04354
Fumaric acid	0.04216
Succinic acid	14.5368
Malic acid	0.46725
Tartaric acid	0.05346
Shikimic acid	0.12474
Citric acid	0.79254
Hydroxycitric acid	0.6102

### *In silico* approach

#### Pass pharmacological potential analysis

Table 7 represents the findings of PASS analysis for all the compounds, which indicate that the compounds have significant antidiabetic activity. The Pa values of citric acid, hydroxyl citric acid, and malic acid were found to be greater than the Pi value of other compounds.

**Table 7:** Predicted PASS results.

**Sl. No**.	**Name of the compound**	**Activity**	**Pa**	**Pi**
1	Citric acid	Antidiabetic	0.648	0.009
2	Fumaric acid	Antidiabetic	0.512	0.021
3	Hydroxycitric acid	Antidiabetic	0.708	0.006
4	Lactic acid	Antidiabetic	0.680	0.007
5	Maleic acid	Antidiabetic	0.512	0.021
6	Malic acid	Antidiabetic	0.639	0.009
7	Malonic acid	Antidiabetic	0.270	0.100
8	Pyruvic acid	Antidiabetic symptomatic	0.228	0.095
9	Shikimic acid	Antidiabetic	0.203	0.160
10	Succinic acid	Antidiabetic	0.440	0.034
11	Tartaric acid	Antidiabetic	0.719	0.005
12	Acarbose	Antidiabetic	0.693	0.007

#### Molecular docking studies

**Figure 6:**
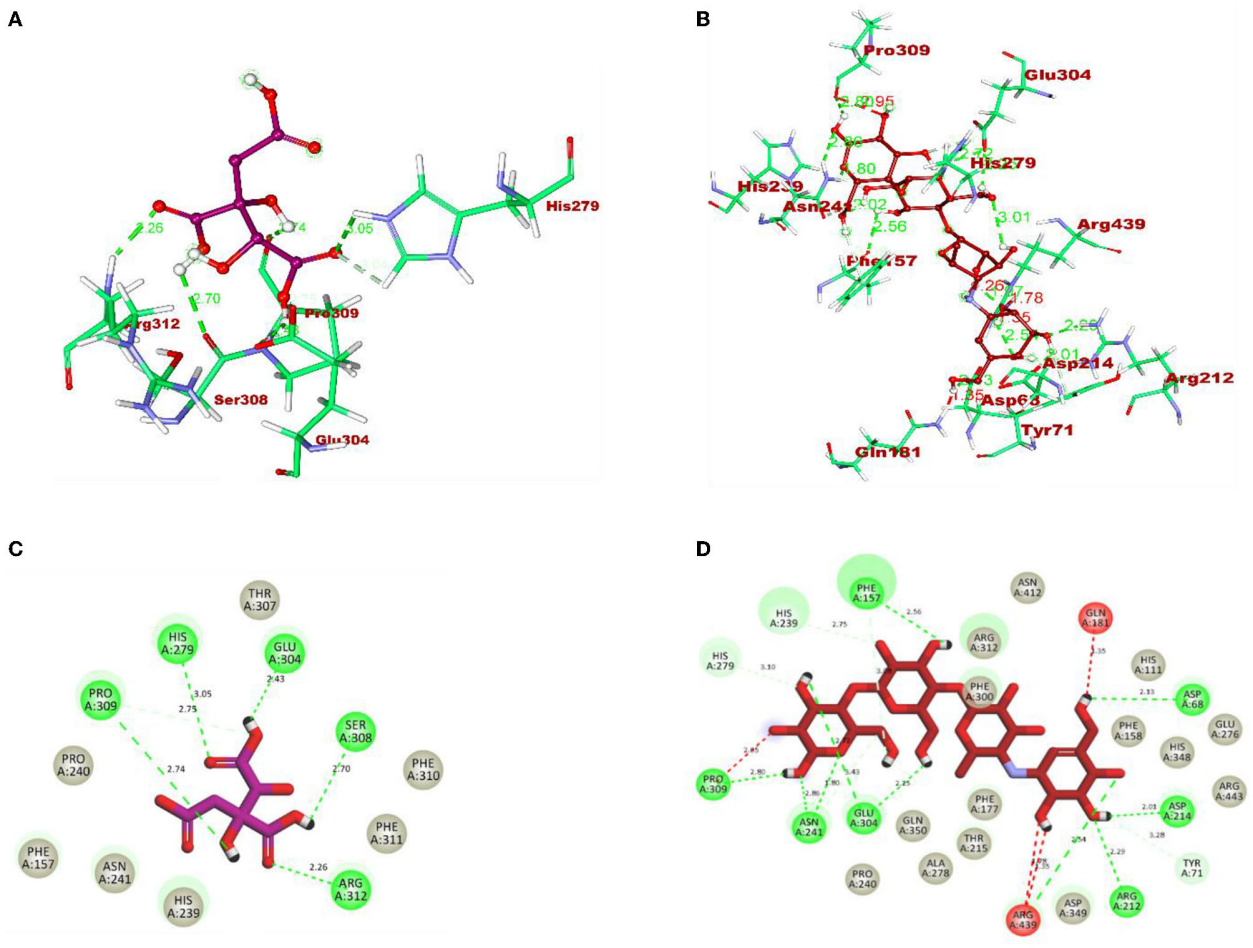
3D representation of ligands in the stick model for hydroxycitric acid and acarbose is given in different colors: **(A)** purple: hydroxycitric acid and **(B)** red: acarbose. The different types of interactions and their respective distance are represented by dotted lines in maroon color and the three-letter amino acids. 2D representation of ligands along with their bounded and non-bounded interactions are represented with their distance as follows: **(C)** purple: hydroxycitric acid and **(D)** red: acarbose.

**Figure 7:**
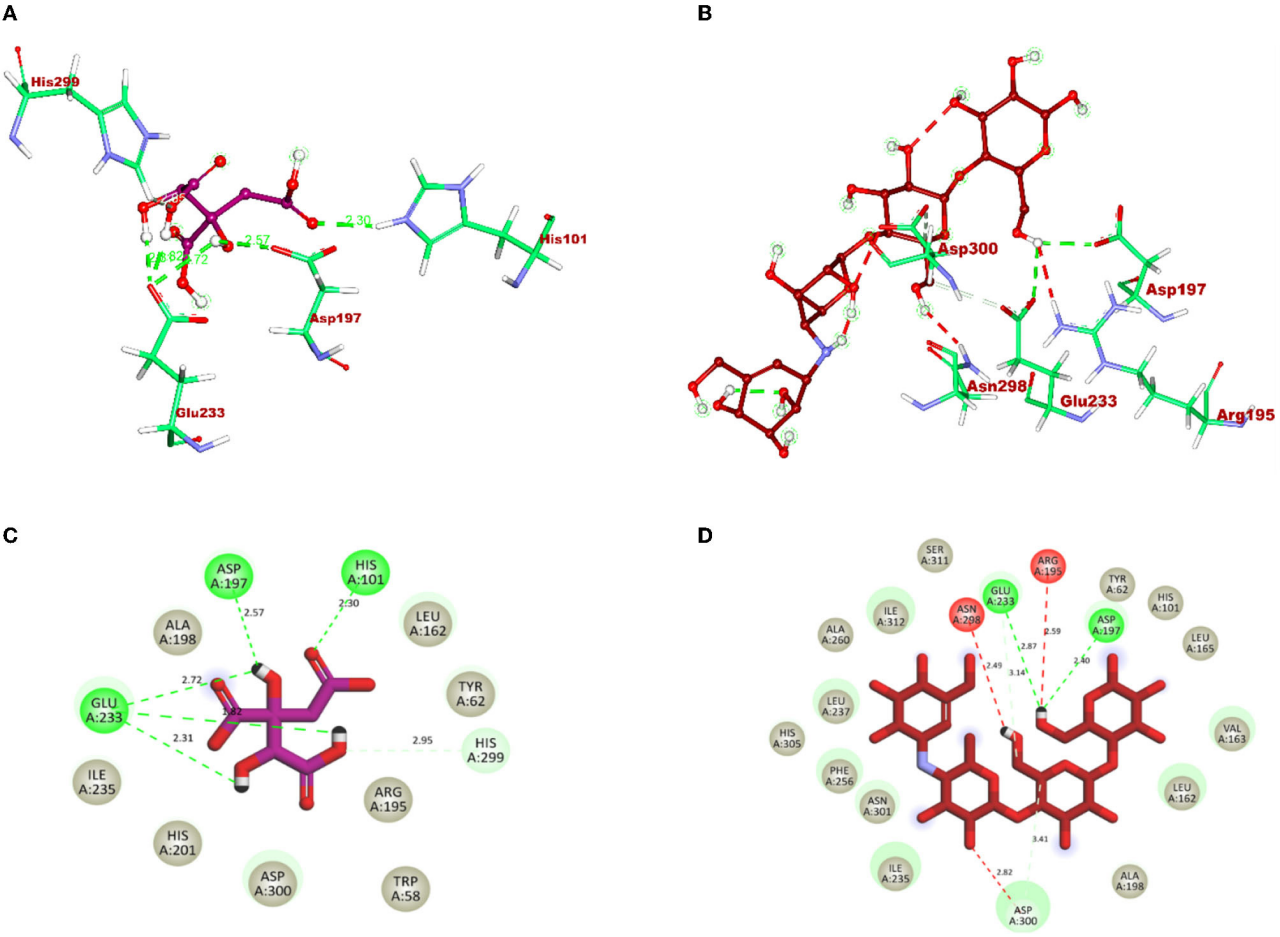
3D representation of ligands in sticks model for hydroxycitric acid and Acarbose is given in different colors: **(A)** purple: hydroxycitric acid and **(B)** red: acarbose. The different types of interactions and their respective distance are represented by dotted lines in maroon color and the three-letter amino acids. 2D representation of ligands along with their bounded and non-bounded interactions are represented with their distance as follows: **(C)** purple: hydroxycitric acid and **(D)** red: acarbose.

After docking, the obtained output was visualized using Discovery Studio to understand the mechanisms of interaction between target proteins and ligands. By 3D visualization, the total number of interactions, the number of hydrogen bonds, and binding affinity of each protein–ligand complex are listed in Table 8. Among all the ligands, hydroxycitric acid was found to have a high docking score of 7 non-bonding interactions, 7 hydrogen bonds, and−6.4 kcal/mol binding affinity with α-glucosidase and 6 non-bonding interactions, 6 hydrogen bonds, and −5.9 kcal/mol with α-amylase. Furthermore, by 2D visualization, the interactions between hydroxy citric acid and the important amino acid residues of α-glucosidase and α-amylase were analyzed, and it was observed that the lead compound interacted with ASN241, ARG312, GLU304, SER308, HIS279, PRO309, and PHE311 of α-glucosidase. The binding mode of the hydroxycitric acid with α-glucosidase was similar to that observed in previous studies (Nivetha et al., 2022; Prabhakaran et al., 2022). Similarly, the results for α-amylase were in accordance with the previous studies (Ganavi et al., 2022; Patil et al., 2022f) (Figure 6). The results showed that GLU233, ASP197 residues of alpha-amylase bound via hydrogen bonds (Figure 7), which were in accordance with previous studies (Ganavi et al., 2022; Patil et al., 2022f).

**Table 8:** Virtual screening of *Levilactobacillus* brevis derivatives against α-glucosidase and α-amylase.

**Sl. No**.	**Name of the compound**	**Binding affinity (kcal/mol)**		**Total no. of non-bonding interactions**		**Total no. of conventional hydrogen bonds**	
		α**-glucosidase**	α**-amylase**	α**-glucosidase**	α**-amylase**	α**-glucosidase**	α**-amylase**
1	Citric acid	−6.1	−5.9	5	3	5	3
2	Fumaric acid	−6.3	−5.4	5	4	5	4
3	Hydroxycitric acid	−6.4	−5.9	7	6	7	6
4	Lactic acid	−5.5	−5.1	4	4	4	3
5	Maleic acid	−6.3	−5.0	5	3	5	3
6	Malic acid	−6.2	−5.2	3	6	3	6
7	Malonic acid	−5.8	−5.1	3	5	3	5
8	Pyruvic acid	−5.5	−5.3	4	2	4	2
9	Shikimic acid	−5.9	−5.6	6	4	6	4
10	Succinic acid	−6.1	−4.9	5	5	5	5
11	Tartaric acid	−6.0	−4.3	7	5	7	5
12	Acarbose	−5.9	−5.4	7	4	6	4

#### Molecular dynamics simulation

After understanding the interaction between the lead compound and target proteins through docking, its simulation was performed to evaluate its dynamics. The difference in interaction between the ligand and targets was unclear, especially with respect to the stability and flexibility of the formed complex (Martiz et al., 2022c; Sajal et al., 2022). Thus, to validate the docking result of hydroxycitric acid with the target proteins, dynamic simulation was conducted using the trajectories in terms of root mean square deviation (RMSD), root mean square fluctuations (RMSFs), radius of gyration (Rg), solvent accessible surface area (SASA), and ligand hydrogen bonds (H-bond number; Pradeep et al., 2022; Patil et al., 2023).

The stability of the complex's conformation at a given time was evaluated using RMSD. Based on the RMSD analysis of the α-glucosidase complex, it can be predicted that both the complex and apoprotein reached the equilibrium after 25 ns. Hydroxycitric acid was observed to remain inside the inhibitor binding site throughout the simulation period. Compared with the interaction with the acarbose complex, the hydroxycitric acid complex reached equilibrium more rapidly, thus showing higher stability (Kumar et al., 2021; Banu et al., 2023). The RMSF analysis of α-amylase depicts that the hydroxycitric acid complex and apoprotein were found to be within the range of 0.20–0.30 nm, whereas the acarbose complex ranged between 0.25 and 0.35 nm. In comparison with that of the acarbose complex, hydroxycitric acid was found to be more stable with minimal fluctuations throughout the simulation (Shivanna et al., 2022; Banu et al., 2023).

The RMSF analysis was performed to examine the binding affinity of the lead compound with its targets. The values for all the residues were measured based on a 100 ns trajectory. In case of α-glucosidase, the plot indicates that the target protein has minimal fluctuations and comparable secondary conformational stability when bound to the compounds, whereas the acarbose complex has more fluctuation, which is the indication of instability inside the inhibitor binding site. Similarly, in case of α-amylase, the RMSF value of hydroxycitric acid complex, acarbose complex, and apoprotein is on par with an almost similar pattern of fluctuations (Maradesha et al., 2022a,b).

The Rg plot analysis was performed to evaluate the possible changes that take place in the structure of protein during the complex formation. On analyzing the Rg plot of α-glucosidase, it is observed that the Rg value of hydroxy citric acid complex and acarbose complex did not change significantly throughout the simulation and kept fluctuating at 2.4 nm, which indicates that the binding site had less influence on the structures. Furthermore, the Rg value of α-amylase along with lead compound and acarbose complex was found to be within the same range of 2.31 nm (Martiz et al., 2022a; Patil et al., 2022c).

**Figure 8:**
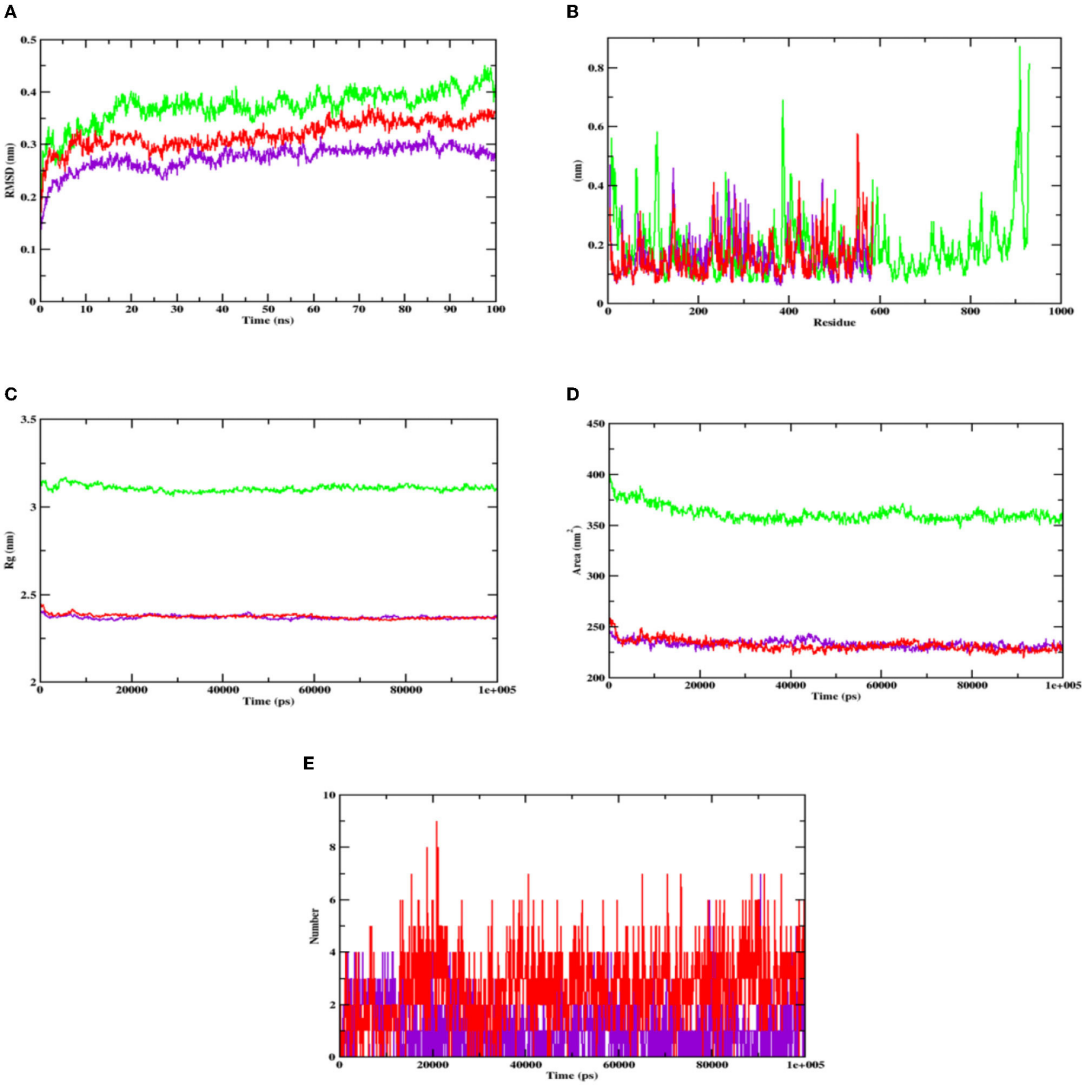
Analysis of RMSD, RMSF, Rg, SASA, and number of hydrogen bonds of hydroxycitric acid (purple) and acarbose (red) bound α-glucosidase complex as well as apoprotein (α-glucosidase: green) at 100 ns. **(A)** Time evolution of the RMSD value of both the complexes along with protein, **(B)** RMSF, **(C)** radius of gyration (Rg), **(D)** SASA, and **(E)** hydrogen bonds.

**Figure 9:**
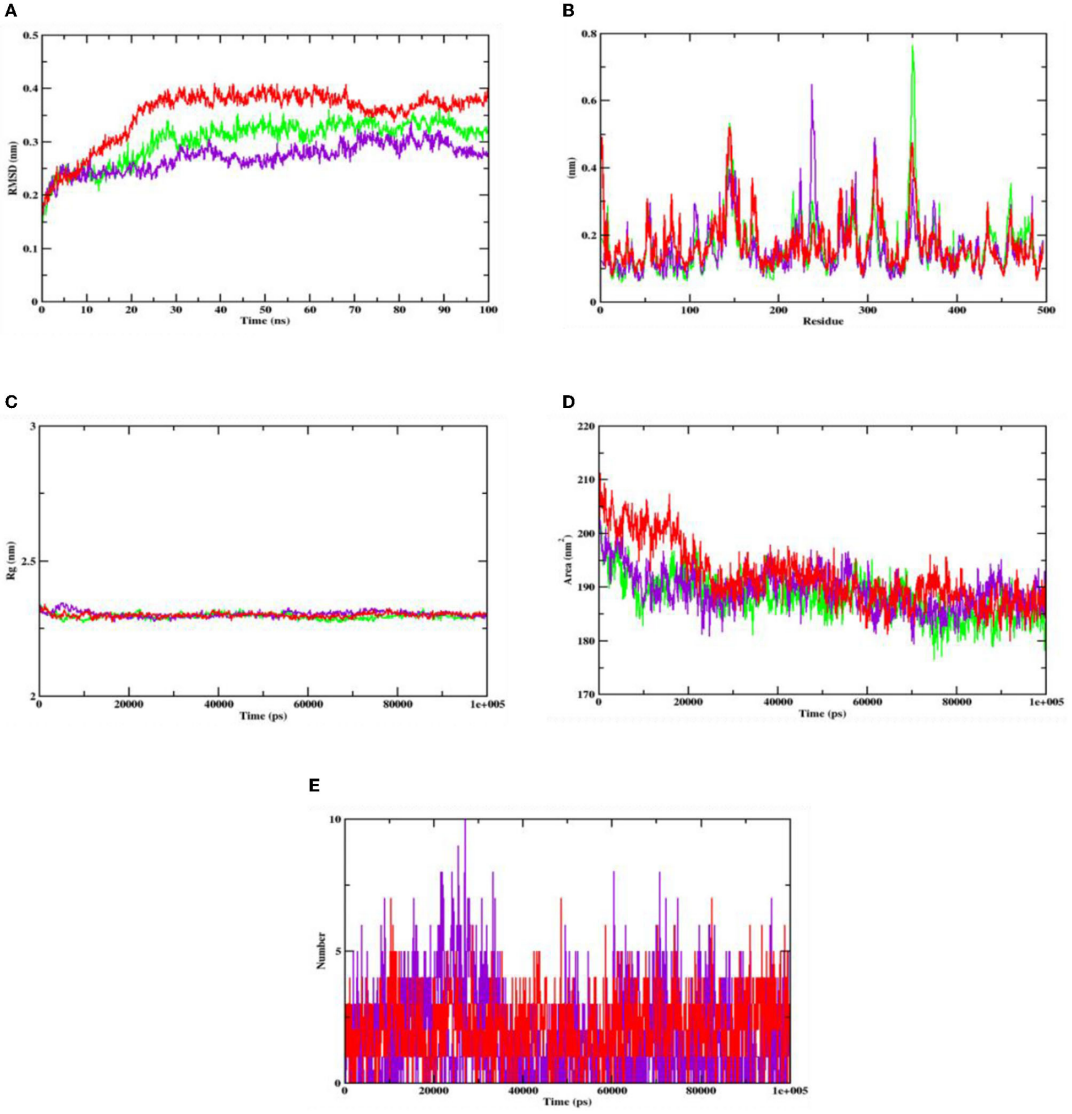
Analysis of RMSD, RMSF, Rg, SASA, and number of hydrogen bonds of hydroxycitric acid (purple) and acarbose (red) bound α-amylase complex as well as apoprotein (α-amylase: green) at 100 ns. **(A)** Time evolution of the RMSD value of both the complexes along with protein, **(B)** RMSF, **(C)** radius of gyration (Rg), **(D)** SASA, and **(E)** hydrogen bonds.

The SASA plot in this study was evaluated to predict the possible conformational changes that take place in the binding region during complex formation. The SASA value of both hydroxycitric acid-α-glucosidase complex and acarbose-α-glucosidase complex and the SASA value of both hydroxycitric acid-α-amylase complex and acarbose-α-amylase complex fluctuated within the range of 190–200 nm^2^. Finally, the ligand hydrogen bonds were analyzed to understand the structural reagreement. In case of α-glucosidase, based on the plot, it can be observed that the complex may have undergone structural modifications. It was observed that hydroxycitric acid formed more H-bonds with the protein during the 100 ns simulation, indicating that the hydroxycitric acid complex was more stable (Maradesha et al., 2022c; Patil et al., 2022f). Concurrently, in the H-bond plot of α-amylase, it was evident that the complex underwent conformational changes. In terms of the ligand hydrogen bonding interactions, acarbose formed fewer hydrogen bonds than hydroxycitric acid (Figures 8, 9).

#### Binding free energy calculations

To gain a deeper understanding of protein–ligand interactions of hydroxycitric acid and acarbose with α-glucosidase and α-amylase as target proteins, binding free energy calculations were considered. The binding free energy analysis revealed that van der Waal's binding energies played a significant role in the formation of complexes. All the binding free energy calculations for hydroxycitric acid were energetically feasible. In comparison, the complexes bound to acarbose had lower binding free energies than those bound to hydroxycitric acid, indicating that their protein–ligand interactions and binding affinities were weaker. The results from binding affinity support the outcomes from molecular docking and dynamics simulation. Additionally, these findings were consistent with previous studies that have performed binding free energy calculations for α-glucosidase and α-amylase (Martiz et al., 2022a). Table 9 summarizes the results of binding free energy calculations obtained using the MM-PBSA technique. Consequently, the study emphasizes the molecular mechanisms underlying protein–ligand interactions and provides details that may aid in the creation of more effective treatments for diabetic nephropathy and other consequences of diabetes.

**Table 9:** Binding free energy values of target proteins complexed with hydroxycitric acid and acarbose.

**Protein-ligand complexes**	**Types of binding free energies**				
	**Van der Waal's energy (kj/mol)**	**Electrostatic energy (kj/mol)**	**Polar solvation energy (kj/mol)**	**SASA energy (kj/mol)**	**Binding energy (kj/mol)**
α-glucosidase-hydroxycitric acid	−220.118	−9.313	96.102	−28.166	−189.1022
α-glucosidase-acarbose	−134.192	−4.813	62.125	−9.310	−90.102
α-amylase-hydroxycitric acid	−211.002	−4.101	29.410	−19.142	−179.001
α-amylase-acarbose	−130.161	−2.106	39.340	−9.564	−87.109

## Discussion

In the present study, the isolation of probiotics from fermented pineapple with the ability to survive in gastrointestinal conditions and the potentiality to inhibit carbohydrate-hydrolyzing enzymes (α-amylase and α-glucosidase) is emphasized. Out of the six single colonies formed, one isolate was selected after the preliminary screening of LAB. Probiotics are bacteria that, when ingested as a food component, frequently have positive effects on health. The presumptive probiotic strain was further evaluated for its ability to withstand different temperatures, pHs, and salt concentrations, and the isolate from fermented pineapple expressed tolerance to all varying conditions. The isolate would have to resist a range of temperatures, pH levels, and salt concentrations to survive once they reach the gut. Furthermore, the aromatic amino acids present in the gut microbiota, which are derived from dietary proteins, lead to the production of phenol (Yadav et al., 2016). Therefore, for a LAB strain to be qualified as a probiotic, it is highly important to have tolerance to phenol. The isolate in this study expressed tolerance up to 0.4% phenol until the last hour of incubation with no significant loss in the cell count, and the results are similar to previous studies (Jena et al., 2013; Abbasiliasi et al., 2017; Jawan et al., 2019). It is critical for a specific LAB strain to be able to withstand some phenol. The LAB strains isolated from the fermented beverage raabadi have a resistance to phenol that ranges from 6.3 to 7.7 log CFU/mL (Yadav and Shukla, 2020). Any food consumed must first endure the harsh environment of the stomach and the small intestine before it can be absorbed into the large intestine. To have beneficial effects of the food consumed on health, probiotics must endure harsh stomach and intestinal conditions. The isolate was evaluated for its ability to withstand extreme acidic and bile conditions, indicating that the isolate can survive at low pH and bile conditions. There are multiple instances reported of gastric and intestinal juice tolerance of probiotic strains isolated from many fermented foods (Nguyen et al., 2019; Kumari et al., 2022a,b). In this study, the survival rate of the RAMULAB49 strain in gastric and intestinal fluids was 6.21 and 7.35 log CFU/mL in the last hour of incubation. The obtained results are on par with previous studies (Zhou et al., 2009; Feng et al., 2017). The non-polar solvent xylene was used to check the surface hydrophobicity of the LAB strain, and the observed affinity was 67.71%. The hydrophobicity supports the adhesion of bacterial cells to the intestinal epithelial cells, and it is directly connected with the autoaggregation ability of the probiotic strain. The autoaggregation of the isolate after 24 h of incubation was >75%, and the obtained results are on par with the findings of Li et al. (2020) and Tuo et al. (2013). The percentage of coaggregation of RAMULAB49 varied with a different pathogen and ranged between 15.72 and 21.98%, and it is an essential part of balancing the ecosystem inside the intestine. Any consumed probiotics will pass through all extreme abdominal conditions in the same pattern of food and get exposed to gastric and intestinal fluids. Therefore, any probiotic needs to show survivability to gastrointestinal fluids. In this study, the survival rate of the isolate was 6.21 and 7.35 log CFU/mL at the incubation periods of 3 and 8 h with pepsin and trypsin, respectively. The observed tolerance was much higher than in previous studies (Vamanu, 2017; Stasiak-Rózańska et al., 2021). The isolate was further checked for its antibacterial activity against 10 food pathogens, and high inhibition was observed by the cell-free supernatant against *M. luteus, P. aeruginosa*, and *S. typhimurium*. The observed inhibition indicates the production of organic acids during the fermentation process and also increases acidic conditions in media. The low pH in the media thus reduces the intercellular pH of the pathogen, which leads to disruptive cell function and cell death (Kivanç et al., 2011). Susceptibility of the probiotics toward antibiotics have underlined importance, and the resistant strain observes all the space to express its benefit to the consumer when probiotics are administered along with antibiotics (combined therapies). The efficiency of the probiotics can also be restricted with antibiotics in case of hypersensitivity to probiotics (Imperial and Ibana, 2016). In the present study, the isolate was resistant to six out of 10 antibiotics and was sensitive to kanamycin, vancomycin, methicillin, and rifampicin. The adhesion of probiotics to the intestinal epithelial cells is highly important, and this action mechanism of bacteria is multifactorial, where it can block the possibility of pathogen adhesion by competing for the binding site of the host cell and also produces antimicrobial components (Monteagudo-Mera et al., 2019). In other words, probiotic adhesion to intestinal epithelial cells leads to immunomodulation (Cammarota et al., 2009). Additionally, it is crucial to understand the isolate's capacity to adhere to gut epithelial cells. This adhesion ability is screened under *in vitro* conditions with chicken crops and buccal epithelial cells. In the present study, the isolate expressed better adhesion with both the epithelial cell. Furthermore, a safety assessment of the isolate was performed, and the isolate was negative for both hemolytic and DNase enzyme activity. Because the isolate lacked DNase, no cell death was noticed, no hemolysis was observed in the hemolytic experiment, and probiotic strains isolated from the curd sample, as shown in the study by Halder et al. (2017), exhibited comparable outcomes. The antioxidant activity of any drug is tremendously important as the ability of the drug to protect the body from damages caused by free radicals induces oxidative stress (Zehiroglu and Sarikaya, 2019). The antioxidant ability of the isolate in this study with DPPH and ABTS was >60 and >74%, respectively, and the results were approximately equal to the MG860 strain used in the study of Kim et al. (2005) and higher than the probiotic strain isolated from kimchi and infant feces (Jang, 2014). The main objective of the present study was to isolate the effective probiotic strain with the ability to inhibit carbohydrate hydrolyzing enzymes. On assessment, the inhibition was found to be 56.19 and 55.69% for α-amylase and α-glucosidase, respectively, with the cell-free supernatant. As the inhibition of carbohydrate hydrolyzing enzyme was predominant in the cell-free supernatant, the organic acids were extracted (LC-MS) from the same, and the inhibitory activity has been further analyzed and confirmed by *in silico* studies. All organic acids were found to have pharmacological potential, whereas the Pa value of citric acid, hydroxycitric acid, and malic acid was found to be greater than that of the other compounds. In this study, the lead compound was found to interact with the following amino acid residues: ASN241, ARG312, GLU304, SER308, HIS279, PRO309, and PHE311 present in α-glucosidase and HIS239 and HIS279 present in α-amylase via hydrogen bond. In molecular docking and binding free energy calculations, binding affinities are expressed as negative values because they represent the free energy change associated with the binding of a ligand molecule to a receptor molecule (Maradesha et al., 2022a; Martiz et al., 2022a). The binding affinity is defined as the difference in energy between the bound and unbound states of the ligand–receptor complex. In other words, it is the energy required to break the ligand–receptor complex formed through molecular docking simulation. A negative binding affinity indicates that the binding process is energetically favorable, meaning that the complex is more stable than the separate ligand and receptor molecules. Therefore, the greater the negative binding affinity, the more energy is required to break the complex, and the greater the stability of the complex (Patil et al., 2022a,b). A similar pattern of interaction was observed with the positive control used, and the obtained results are in accordance with the previous studies conducted by Patil et al. (2022c,f) and Prabhakaran et al. (2022) with the same target proteins. Inhibition of these enzymes effectively manages blood glucose levels with negligible side effects.

## Conclusion

The present study demonstrated that fermented pineapple contains *Levilactobacillus brevis* RAMULAB49, a probiotic with antidiabetic properties. This study is the first to obtain LAB isolates with antidiabetic potential from fermented pineapple. The safety and high potential for survival in challenging gut environments of the *Levilactobacillus brevis* RAMULAB49 have been established. These results aid in the development of a potentially effective and affordable antidiabetic supplement with minimal adverse effects when compared to any other synthetic medicine. The autoaggregation, hydrophobicity, and adhesion to epithelial cells of the RAMULAB49 strain were also shown to be remarkable. The expression of inhibitory activity against the enzymes α-glucosidase and α-amylase was evaluated *in vitro* and *in silico*. Both the cell-free extract and the intact cells displayed inhibitory capacity, demonstrating that the necessary elements are present in both. However, the cell-free supernatant showed higher inhibition. Therefore, the inhibitory action of this probiotic against the enzymes is strongly supported by an *in silico* approach using organic acid from cell-free supernatant, whereas citric acid, hydroxycitric acid, and malic acid were found to have greater pharmacological potential than the other organic acid from the cell-free supernatant. This research demonstrates the presence of LAB in fermented pineapple, which has potential antidiabetic activities. Therefore, the probiotic strain can be utilized as an efficient antidiabetic therapy and combined with an appropriate dietary composition for improving human health.

## Data availability statement

The datasets presented in this study can be found in online repositories. The names of the repository/repositories and accession number(s) can be found in the article/supplementary material.

## Author contributions

RR planned and conceptualized the manuscript and was involved in supervision and editing. RM, CK, SH, and AP were involved in data analysis and method development. MK, NA, FA, SA, and NS contributed to the original draft preparation and writing. All authors have read and agreed to the published version of the manuscript.
